# Primary and booster vaccination in Latin American children with a DTPw-HBV/Hib combination: a randomized controlled trial

**DOI:** 10.1186/1471-2334-10-297

**Published:** 2010-10-15

**Authors:** Felix Espinoza, Miguel Tregnaghi, Angela Gentile, Katia Abarca, Javier Casellas, Alix Collard, Inge Lefevre, Jeanne-Marie Jacquet

**Affiliations:** 1 Universidad Nacional Autonoma de Leon, Leon, Nicaragua; 2 Centro de Desarrollo de Proyectos Avanzados, Cordoba, Argentina; 3 Hospital de Niños Ricardo Gutierrez, Buenos Aires, Argentina; 4 Pontificia Universidad Católica de Chile, Santiago, Chile; 5 GlaxoSmithKline Biologicals, Buenos Aires, Argentina and Wavre, Belgium

## Abstract

**Background:**

Diphtheria-tetanus-whole-cell pertussis (DTPw)-based combination vaccines are an attractive option to rapidly achieve high coverage and protection against other important pathogens, such as hepatitis B virus (HBV) and *Haemophilus influenzae *type B (Hib). To ensure adequate antigen supply, GlaxoSmithKline Biologicals has introduced a new DTPw antigen source and developed a new DTPw-HBV/Hib combination vaccine containing a reduced amount of Hib polyribosylribitol phosphate (PRP). This study was undertaken to compare the immunogenicity and reactogenicity of this new DTPw-HBV/Hib vaccine with a licensed DTPw-HBV/Hib vaccine (*Tritanrix*™-HBV/Hib).

**Methods:**

This was a randomized, partially-blind, multicenter study in three countries in Latin America (Argentina, Chile and Nicaragua). Healthy children received either the new DTPw-HBV/Hib vaccine (1 of 3 lots; n = 439; double-blind) or Tritanrix™-HBV/Hib (n = 146; single-blind) co-administered with oral poliovirus vaccine (OPV) at 2, 4 and 6 months, with a booster dose at 18-24 months.

**Results:**

One month after the end of the 3-dose primary vaccination course, the new DTPw-HBV/Hib vaccine was non-inferior to Tritanrix™-HBV/Hib in terms of seroprotection/vaccine response rates for all component antigens; ≥97.3% and ≥93.9% of subjects in the two groups, respectively, had seroprotective levels of antibodies against diphtheria, tetanus, hepatitis B and Hib and a vaccine response to the pertussis component. Persistence of antibodies against all vaccine antigens was comparable between groups, with marked increases in all antibody concentrations after booster administration in both groups. Both vaccines were generally well-tolerated as primary and booster doses.

**Conclusions:**

Results confirm the suitability of this new DTPw-HBV/Hib vaccine comprising antigens from a new source and a reduced PRP content for inclusion into routine childhood vaccination programs.

**Trial registration:**

http://www.clinicaltrials.gov NCT00332566

## Background

Combined diphtheria-tetanus-whole cell pertussis (DTPw) vaccines remain the cornerstone of childhood vaccination programs in Latin America and many other parts of the world [1]. The addition of new antigens to existing vaccines with established high coverage rates is an efficient method of rapidly achieving high coverage and protection against other important pathogens with minimum impact on vaccination logistics and cost [2-4]. Hepatitis B (HBV) and *Haemophilus influenzae *type b (Hib) infections remain endemic in many parts of the world, causing disease that can readily be prevented by vaccination [5,6]. Although universal vaccination of infants against HBV and Hib has been recommended by the World Health Organization (WHO) since 1992 and 1996, respectively [7-9], uptake of both vaccines is incomplete. Lack of appropriate combination vaccines and difficulties with vaccine supply have been identified as key factors contributing to this slow uptake [10].

*Tritanrix*™-HBV (GlaxoSmithKline [GSK] Biologicals, Rixensart, Belgium), a combined DTPw and hepatitis B vaccine, has been available since the mid-1990s [11]. This vaccine can be mixed with a conjugated Hib vaccine (*Hiberix™; *GSK Biologicals) and administered as a single injection (*Tritanrix*™-HBV/Hib) [11,12]. In order to address the increasing international demand for DTPw-based combination vaccines, GSK Biologicals has recently introduced a new source of DTPw antigens and has developed a new DTPw-HBV/Hib combination vaccine containing a reduced amount of Hib capsular polyribosylribitol phosphate (PRP) (2.5 μg per 0.5 mL dose instead of the 10 μg PRP contained in *Tritanrix*™-HBV/Hib). DTPw-based combination vaccines with reduced PRP antigen content have been shown to be non-inferior to those with higher PRP antigen content in terms of immune response to all component antigens after primary and booster vaccination [13-18].

The ability to co-administer DTPw-based combination vaccines with other routine vaccines would be convenient for both medical staff and vaccine recipients. Studies have shown the potential for co-administration of combined DTPw-based combination vaccines with other pediatric vaccines, including rotavirus vaccine and oral poliovirus vaccine (OPV) [19]. This study was undertaken to assess the immunogenicity and reactogenicity of GSK Biological's new DTPw-HBV/Hib vaccine compared with *Tritanrix*™-HBV/Hib when co-administered with OPV in healthy Latin American infants at 2, 4 and 6 months. Antibody persistence and immune response to a booster dose at 18-24 months of age was also assessed.

## Methods

### Study design and subjects

This was a randomized, partially-blind, multicenter study in three countries in Latin America (Argentina, Chile and Nicaragua) between August 2004 and September 2005. The study was approved by the appropriate local ethics committees and conducted in accordance with the Declaration of Helsinki and Good Clinical Practice guidelines. Healthy male and female infants born after a normal gestation period (between 36-42 weeks) were enrolled for first vaccination at 6-10 weeks of age. In Argentina, mothers of prospective participants were screened prenatally for the presence of hepatitis B virus surface antigen (HBsAg). Infants born to anti-HBsAg seropositive mothers were ineligible for study participation, but were offered HBV vaccination at birth. Other exclusion criteria included: previous diphtheria, tetanus, pertussis, hepatitis B, Hib or polio vaccination or disease; Bacille Calmette-Guérin vaccine given later than the first 2 weeks of life; major congenital defects or serious chronic illness; a history of allergic disease or reactions likely to be exacerbated by any component of the vaccine; a history of neurological disease, including previous seizures; any immune deficiency and prior or planned use of any blood products, immunoglobulins or immunosuppressive therapy; and acute illness at the time of enrolment. Written informed consent was obtained from the parent or guardian of each infant prior to study entry.

Subjects were randomized in a 1:1:1:1 ratio to receive either one of three lots of the new DTPw-HBV/Hib vaccine or the control vaccine (*Tritanrix*™-HBV/Hib) co-administered with OPV at 2, 4 and 6 months of age. The three different production lots of the new DTPw-HBV/Hib vaccine were administered in a double-blind manner. Blinding was single-blind with regard to the control vaccine group since the appearance of the two vaccines was not exactly the same. Subjects in Nicaragua and Argentina who had completed the 3-dose primary vaccination course were invited to receive a booster dose of the same DTPw-HBV/Hib combination vaccine at 18-24 months of age. The booster study took place between June and October 2006.

### Objectives

The two co-primary objectives of this study were to demonstrate both lot-to-lot-consistency of the new DTPw-HBV/Hib vaccine and non-inferiority of the new DTPw-HBV/Hib vaccine to *Tritanrix*™-HBV/Hib in terms of antibody response one month after the third primary vaccine dose. For all vaccine antigens, antibody persistence was assessed prior to booster administration and antibody responses were assessed one month after administration of the booster dose.

### Vaccines

All vaccines were manufactured by GSK Biologicals. The diphtheria and tetanus antigens were sourced form GSK Biologicals Korlatolt Felelossegu Tarsasag (Hungary) and Pw was obtained from Commonwealth Serum Laboratory Ltd. (Australia) for the new DTPw-HBV/Hib vaccine. Both vaccines contained ≥30 international units (IU) diphtheria toxoid, ≥60 IU tetanus toxoid, ≥4 IU killed *Bordetella pertussis*, and 10 μg HBsAg. The new DTPw-HBV/Hib vaccine contained 2.5 μg of Hib capsular PRP polysaccharide conjugated to 5-10 μg tetanus toxoid. *Tritanrix*™-HBV/Hib contained 10 μg of Hib capsular PRP polysaccharide conjugated to 20-40 μg tetanus toxoid. Both vaccines were administered as a 0.5 mL intramuscular injection into the anterolateral region of the left thigh. All subjects received OPV concomitantly with each primary dose.

### Serology

Blood samples were collected prior to administration of the first vaccine dose and one month after completion of the 3-dose primary course. Blood samples were also taken before and one month after administration of the booster dose. Samples were stored at -20°C until testing at GSK Biologicals' central laboratory in Rixensart, Belgium. Concentrations of antibodies against all vaccine antigens were determined by enzyme-linked immunosorbent assay (ELISA). Assay cut-offs were 0.1 IU/mL for diphtheria and tetanus, 15 IU/mL for *Bordetella pertussis *toxin (BPT), 10 mIU/mL for HBsAg and 0.15 μg/mL for PRP. Pre-booster samples that were negative by ELISA were retested with the more sensitive in vitro neutralization assay on Vero cells with a cut-off of 0.016 mIU/ml.

With the exception of anti-BPT, antibody concentrations equal to or greater than the assay cut-off were considered to be protective. As no correlate of protection is established for *B. pertussis*, a vaccine response to this vaccine component was defined. For primary vaccination, a pertussis vaccine response was defined as the appearance of anti-BPT antibodies in initially seronegative subjects, or the presence of a post-vaccination antibody concentration greater than or equal to the initial pre-vaccination concentration. A booster response against the pertussis component was defined as the appearance of anti-BPT antibodies in pre-booster seronegative subjects, or at least a 2-fold increase in anti-BPT antibody concentration in subjects seropositive prior to boosting.

### Reactogenicity and safety

Parents/guardians used diary cards to record the occurrence of solicited local (pain, redness and swelling at the site of injection) and general (drowsiness, fever [defined as rectal temperature ≥38°C], irritability and loss of appetite) symptoms during a 4-day follow-up period after each vaccination. Intensity of symptoms was graded on a scale of 0-3. Grade 3 symptoms were defined as follows: for local pain as crying when limb is moved or a spontaneously painful limb; for local redness and swelling as diameter > 20 mm; for fever as rectal temperature > 40.0°C; and for all other symptoms as preventing normal daily activities. All other (unsolicited) adverse events were recorded for a period of 30 days after each vaccination. Serious adverse events (SAEs) were recorded during the entire study period. All solicited local symptoms were considered causally related to vaccination. Causality of solicited general symptoms, unsolicited symptoms and SAEs was assessed by the investigator.

### Statistical analysis

The target sample size was 500 evaluable subjects for immunogenicity (125 subjects per vaccine group). Considering that approximately 15% of enrolled subjects might withdraw or be unevaluable for immunogenicity; the target enrolment size was 600 subjects (150 subjects per vaccine group).

Analysis of immunogenicity was performed on the according-to-protocol (ATP) cohort and the analysis of safety was performed on the total vaccinated cohort.

Seroprotection rates against diphtheria, tetanus, HBsAg and PRP and vaccine response rates to BPT were calculated with 95% CIs. Standardized asymptotic 95% CIs for the differences in seroprotection/vaccine response rates between the pooled DTPw-HBV/Hib groups and the *Tritanrix*™-HBV/Hib group one month after the third primary vaccine dose were computed. The response to the new DTPw-HBV/Hib vaccine was considered non-inferior to that of the control vaccine if the upper limit of the 95% CI for the absolute difference between groups was lower than 10% for D, T, PRP and HBs. GMCs with 95% CI were calculated by taking the log-transformation of individual concentrations and calculating the anti-log of the mean of these transformed values. Antibody concentrations below the assay cut-off were given an arbitrary value of half the cut-off value for the purpose of GMC calculation. For, D, T, PRP and HBs antigens, 95% CIs for the GMC ratio (control over pooled DTPw-HBV/Hib groups) one month after primary vaccination were computed using an ANOVA model on the logarithm in base 10 (log_10_) transformation of the concentrations using the vaccine group as covariate, while for pertussis, the 95% CI for the GMC ratio was computed using an ANCOVA model with the vaccine group and the log_10 _concentrations prior to vaccination as covariates.

The incidence of solicited local and general symptoms (any or Grade 3 intensity) was calculated with exact 95% CI. Differences between groups were compared using two-sided Fisher exact tests. P-values <0.05 were considered as possibly indicative of a statistically significant difference between groups.

The statistical analyses were performed using the Statistical Analysis Systems (SAS) version 8.2 on Windows XP Professional and StatXact-5 procedure on SAS.

## Results

**Figure 1:**
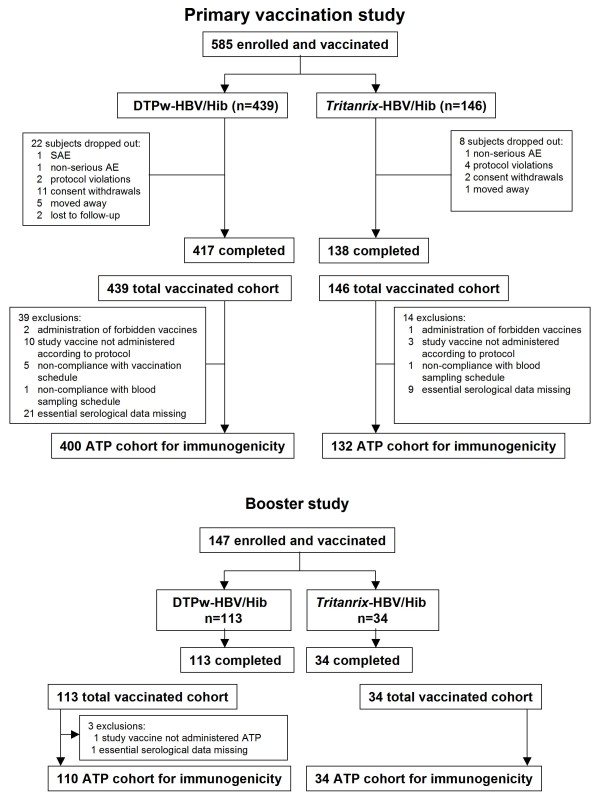
Subject disposition

A total of 585 subjects were enrolled and received primary vaccination (439 in the DTPw-HBV/Hib group and 146 in the *Tritanrix*™-HBV/Hib group). Of these, 147 subjects received booster vaccination at 18-24 months of age (113 and 34 subjects in the two groups, respectively). The relatively low proportion of subjects evaluated for booster vaccination is due to only two countries out of three (Argentina and Nicaragua) participating in the booster study. The mean (±SD) age of the total cohort at the time of first vaccination was 7.8 (±1.12) weeks, 52.0% of subjects were male and 56.6% were American Hispanic. The mean (±SD) age at the time of the booster dose was 18.9 (±0.7) months and 59.9% of subjects were male. The population was predominantly American Hispanic (78.2%); the other subjects were White/Caucasian. No clinically significant differences in demography were seen between groups. Subject disposition is summarized in Figure 1.

### Immunogenicity

Seroprotection/vaccine response rates and antibody GMCs for all vaccine antigens are shown in Table 1. The new DTPw-HBV/Hib vaccine was found to be non-inferior to *Tritanrix*™-HBV/Hib in terms of seroprotection against diphtheria, tetanus, HBs and PRP antigens following primary vaccination. One month after completion of the 3-dose primary course, 99.0% of subjects in the DTPw-HBV/Hib group and 100% of those in the *Tritanrix*™-HBV/Hib group had anti-PRP antibody concentrations ≥0.15 μg/mL, with anti-PRP antibody concentrations ≥1.0 μg/mL in 97.3% and 100% of subjects in the two groups, respectively. At least 97.7% of subjects in the DTPw-HBV/Hib group and 93.9% of those in the *Tritanrix*™-HBV/Hib group had seroprotective levels of antibodies against diphtheria, tetanus and hepatitis B and a vaccine response to the pertussis component at this time. Post-primary vaccination GMCs for anti-diphtheria and anti-HBs antibodies were significantly higher (non-overlapping CI) in the DTPw-HBV/Hib group than in the *Tritanrix*™-HBV/Hib control group, while anti-PRP GMC was significantly higher in the *Tritanrix*™-HBV/Hib group than in the DTPw-HBV/Hib group (21.4 and 12.5 μg/mL, respectively; non-overlapping CI).

**Table 1:** Seroprotection (SP), vaccine response (VR) rates and GMCs one month post-primary and pre- and one month post-booster vaccination (ATP immunogenicity cohort)

	Post-Primary			Pre-Booster			Post-Booster		
Group	N	%SP/VR (95% CI)	GMC (95% CI)	N	%SP (95% CI)	GMC (95% CI)	N	%SP/BR (95% CI)	GMC (95% CI)
**Anti-diphtheria (≥0.1 IU/mL)**									
DTPw-HBV/Hib	398	97.7 (95.8, 99.0)	2.093§ (1.851, 2.366)	109	81.7 (73.1, 88.4)	0.263 (0.218, 0.316)	110	100 (96.7, 100)	6.755 (5.726, 7.970)
*Tritanrix*™-HBV/Hib	132	93.9 (88.4, 97.3)	1.350§ (1.077, 1.691)	33	75.8 (57.7, 88.9)	0.214 (0.153, 0.300)	34	100 (89.7, 100)	4.974 (3.197, 7.738)
**Anti-tetanus (≥0.1 IU/mL)**									
DTPw-HBV/Hib	399	99.7 (98.6, 100)	6.679 (6.084, 7.333)	110	98.2 (93.6, 99.8)	0.652 (0.555, 0.767)	110	100 (96.7, 100)	18.555 (16.406, 20.984)
*Tritanrix*™-HBV/Hib	131	100 (97.2, 100)	5.884 (4.964, 6.974)	34	100 (89.7, 100)	0.748 (0.564, 0.991)	34	100 (89.7, 100)	15.430 (12.205, 19.507)
**Anti-HBs (≥10 mIU/mL)**									
DTPw-HBV/Hib	400	99.8 (98.6, 100)	2417.5§ (2127.3, 2747.3)	110	94.5 (88.5, 98.0)	126.9 (98.1, 164.2)	110	99.1 (95.0, 100)	19247.5 (14422.5, 25686.6)
*Tritanrix*™-HBV/Hib	132	97.7 (93.5, 99.5)	1319.3§ (1035.8, 1680.3)	34	94.1 (80.3, 99.3)	97.3 (65.5, 144.3)	34	97.1 (84.7, 99.9)	14366.8 (7612.5, 27114.2)
**Anti-PRP (≥0.15 μg/mL)**									
DTPw-HBV/Hib	400	99.0 (97.5, 99.7)	12.530§ (11.180, 14.043)	110	95.5 (89.7, 98.5)	1.006§ (0.768, 1.319)	110	100 (96.7, 100)	34.364§ (27.563, 42.843)
*Tritanrix*™-HBV/Hib	132	100 (97.2, 100)	21.393§ (17.971, 25.467)	34	100 (89.7, 100)	2.059§ (1.357, 3.123)	34	100 (89.7, 100)	66.616§ (43.734, 101.468)
**Anti-PRP (≥1.0 μg/mL)**									
DTPw-HBV/Hib	400	97.3 (95.1, 98.6)	-	110	46.4 (36.8, 56.1)	-	110	99.1 (95.0, 100)	-
*Tritanrix*™-HBV/Hib	132	100 (97.2, 100)	-	34	79.4 (62.1, 91.3)	-	34	100 (89.7, 100)	-
**Anti-BPT (VR or BR)**									
DTPw-HBV/Hib	394*	97.9 (96.0, 99.1)	82.6 (76.7, 89.1)	110		10.9 (9.8, 12.1)	109	99.1 (95.0, 100)	105.1 (93.6, 118.0)
*Tritanrix*™-HBV/Hib	131	95.4 (90.3, 98.3)	90.5 (77.7, 105.5)	33		11.1 (9.1, 13.7)	34#	100 (89.5, 100)	128.4 (99.2, 166.3)

N = number of subjects with available results; *390 subjects for vaccine response; #33 subjects for booster response

Vaccine (VR) response to the pertussis component defined as the appearance of antibody concentration ≥15 EL.U/mL in subjects seronegative at pre-vaccination or maintenance of pre-vaccination antibody concentrations one month after the third dose in initially seropositive subjects prior to primary vaccination.

Booster (BR) response to the pertussis component defined as the appearance of antibody concentration ≥15 EL.U/mL in subjects seronegative at pre-booster or post-booster antibody concentration two-fold higher than pre-booster antibody concentrations in initially seropositive subjects prior to the booster vaccination.

%SP = percentage of subjects who achieve seropositivity as is described for each antigen; GMC = geometric mean antibody concentration; 95%CI = 95% confidence interval; § non-overlapping CI

Prior to booster administration at 18-24 months, anti-PRP antibody concentrations remained ≥0.15 μg/mL in 95.5% of subjects in the DTPw-HBV/Hib and 100% of those in the *Tritanrix*™-HBV/Hib group. More than 81.7% and 75.8% of subjects in the two groups, respectively, still had seroprotective levels of antibodies to diphtheria (by ELISA), tetanus and hepatitis B at this time, with 32.7% and 36.4% of subjects remaining seropositive for anti-BPT antibodies (data not shown). Testing of seronegative samples by the Vero cell neutralisation assay showed a global seroprotection rate against diphtheria of 90.5% and 82.4% in the two groups, respectively. No differences in pre-booster antibody GMCs were seen between the two groups, with the exception of a significantly higher anti-PRP GMC in the *Tritanrix*™-HBV/Hib group (2.1 compared with 1.0 μg/mL in the DTPw-HBV/Hib group; non-overlapping CI).

A marked booster response to all vaccine antigens was observed in both groups. One month after booster administration, all subjects had anti-PRP antibody concentrations ≥0.15 μg/mL, with anti-PRP antibody concentrations ≥1.0 μg/mL in all but one subject in the DTPw-HBV/Hib vaccine group. At least 99.1% of subjects in the DTPw-HBV/Hib group and 97.1% of those in the *Tritanrix*™-HBV/Hib group had seroprotective levels of antibodies against diphtheria, tetanus and hepatitis B and a booster response to the pertussis component following booster administration. Post-booster GMCs did not appear to differ between the two groups, with the exception of anti-PRP GMC which remained significantly higher in the *Tritanrix*™-HBV/Hib group than in the DTPw-HBV/Hib group (66.6 and 33.4 μg/mL, respectively; non-overlapping CI).

### Reactogenicity and safety

The overall incidence of solicited local and general symptoms following primary and booster doses is shown in Table 2. Pain was the most frequent local solicited symptom in both groups. Grade 3 pain appeared to be more common in the DTPw-HBV/Hib group than in the *Tritanrix*™-HBV/Hib group after both primary and booster doses (21.8% and 15.4% in the two groups, respectively, following primary vaccination; non-overlapping CI, and 43.4% and 23.5%, respectively, after the booster dose). Irritability and fever were the most common general solicited symptoms. One subject in each group withdrew due to Grade 3 irritability following administration of the first primary dose. Irritability occurred more frequently after the booster dose in the DTPw-HBV/Hib group than in the *Tritanrix*™-HBV/Hib group (71.7% versus 47.1%, respectively). The incidence of fever (rectal temperature ≥38.0°C) was higher in the DTPw-HBV/Hib group than in the *Tritanrix*™-HBV/Hib group following both primary and booster vaccination (56.9% versus 46.7%; non-overlapping CI and 70.8% versus 58.8%, respectively). However, the incidence of rectal temperature ≥40.0°C was low and similar in both groups after all doses. Very few subjects required medical advice for solicited symptoms following either primary or booster doses (Table 2).

**Table 2:** Incidence (% [95% CI]) of solicited local and general symptoms during the 4 days following primary (overall/dose) and booster vaccination (Total vaccinated cohort)

	Primary doses		Booster dose	
Solicited symptom	DTPw-HBV/Hib (N = 1274)	*Tritanrix*™-HBV/Hib (N = 422)	DTPw-HBV/Hib (N = 113)	*Tritanrix*™-HBV/Hib (N = 34)
Pain				
Any	73.4 (70.9, 75.8)	69.4 (64.8, 73.8)	90.3 (83.2, 95.0)	79.4 (62.1, 91.3)
Grade 3	21.8 (19.6, 24.2)§	15.4 (12.1, 19.2)§	43.4 (34.1, 53.0)	23.5 (10.7, 41.2)
MA	0.5 (0.2, 1.1)	0.5 (0.1, 1.7)	0 (0.0, 3.2)	0 (0.0, 10.3)
Redness				
Any	53.1 (50.4, 55.9)§	44.1 (39.3, 49.0)§	48.7 (39.2, 58.3)	44.1 (27.2, 62.1)
> 20 mm	17.3 (15.2, 19.5)	12.8 (9.8, 16.4)	14.2 (8.3, 22.0)	11.8 (3.3, 27.5)
MA	0.6 (0.3, 1.2)	0 (0.0, 0.9)	0 (0.0, 3.2)	0 (0.0, 10.3)
Swelling				
Any	51.1 (48.3, 53.9)	46.4 (41.6, 51.3)	64.6 (55.0, 73.4)	38.2 (22.2, 56.4)
> 20 mm	21.7 (19.4, 24.0)	23.2 (19.3, 27.6)	28.3 (20.2, 37.6)	17.6 (6.8, 34.5)
MA	0.7 (0.3, 1.3)	0.2 (0.0, 1.3)	0.9 (0.0, 4.8)	0 (0.0, 10.3)
Drowsiness				
Any	44.3 (41.6, 47.1)	44.1 (39.3, 49.0)	39.8 (30.7, 49.5)	17.6 (6.8, 34.5)
Grade 3	1.5 (0.9, 2.3)	0.9 (0.3, 2.4)	2.7 (0.6, 7.6)	0 (0.0, 10.3)
MA	0.4 (0.1, 0.9)	0.2 (0.0, 1.3)	0 (0.0, 3.2)	0 (0.0, 10.3)
Rectal temperature				
Any	56.9 (54.1, 59.6)§	46.7 (41.8, 51.6)§	70.8 (61.5, 79.0)	58.8 (40.7, 75.4)
> 38.0°C	56.9 (54.1, 59.6)§	46.7 (41.8, 51.6)§	38.1 (29.1, 47.7)	14.7 (5.0, 31.1)
> 38.5°C	19.6 (17.5, 21.9)	17.5 (14.0, 21.5)	21.2 (14.1, 29.9)	5.9 (0.7, 19.7)
> 39.0°C	6.9 (5.6, 8.4)	5.0 (3.1, 7.5)	1.8 (0.2, 6.2)	2.9 (0.1, 15.3)
> 39.5°C	2.8 (2.0, 3.9)	3.1 (1.7, 5.2)	0.9 (0.0, 4.8)	0.0 (0.0, 10.3)
> 40.0°C	0.5 (0.2, 1.0)	0.7 (0.1, 2.1)	0.0 (0.0, 3.2)	0.0 (0.0, 10.3)
MA	1.2 (0.7, 1.9)	0.5 (0.1, 1.7)	0.0 (0.0, 3.2)	0.0 (0.0, 10.3)
Irritability				
Any	67.0 (64.4, 69.6)	65.2 (60.4, 69.7)	71.7 (62.4, 79.8)	47.1 (29.8, 64.9)
Grade 3	5.0 (3.9, 6.4)	6.6 (4.5, 9.4)	9.7 (5.0, 16.8)	0 (0.0, 10.3)
MA	0.7 (0.3, 1.3)	0.7 (0.1, 2.1)	0 (0.0, 3.2)	0 (0.0, 10.3)
Loss of appetite				
Any	29.9 (27.4, 32.5)	26.1 (21.9, 30.5)	42.5 (33.2, 52.1)	29.4 (15.1, 47.5)
Grade 3	0.8 (0.4, 1.4)	0.5 (0.1, 1.7)	4.4 (1.5, 10.0)	0 (0.0, 10.3)
MA	0.3 (0.1, 0.8)	0.2 (0.0, 1.3)	0 (0.0, 3.2)	0 (0.0, 10.3)

N = number of doses; MA = medical advice sought for the symptom; § non-overlapping CI

The incidence of unsolicited symptoms considered related to vaccination did not differ between groups (17.7% of the doses administered in the DTPw-HBV/Hib group and 17.1% in the *Tritanrix*™-HBV/Hib group after primary vaccination and none were reported in either of the groups after the booster dose). The most common unsolicited symptom considered related to vaccination after primary vaccination was injection site reactions in both groups. Injection site induration was reported following 15.9% and 15.6% of the vaccine doses in the DTPw-HBV/Hib and in the *Tritanrix*™-HBV/Hib groups respectively. Two SAEs considered related to vaccination were reported during the primary course. Both of these events occurred in the DTPw-HBV/Hib group and required hospitalization, but resolved without sequelae. One subject who presented persistent crying on the day of administration of the first primary dose remained to complete the 3-dose primary vaccination course. The other subject experienced erythema and induration at the vaccine injection site six days after vaccination. The subject was diagnosed as having left thigh abscess and was treated. Following discharge the subject recovered, but was subsequently withdrawn from the study. No SAEs considered related to vaccination were reported after booster administration.

## Discussion

Clinical experience shows DTPw-HBV/Hib combination vaccines to be an efficient and reliable method of implementing WHO recommendations for controlling diphtheria, tetanus, pertussis, hepatitis B and Hib infections on a worldwide basis [11]. For the new vaccine evaluated in this study, new antigen sources and reduced antigen content were used to ensure continued vaccine supply for global mass vaccination campaigns. This DTPw-HBV/Hib combination vaccine is based on the same formulation as the licensed *Tritanrix*™-HBV/Hib vaccine [12,20], but containing diphtheria, tetanus and pertussis antigens from an alternative source and a lower amount of the PRP capsular polysaccharide component of Hib (2.5 instead of 10 μg/mL per 0.5 ml dose). The study was designed and powered for immunogenicity comparison in primary vaccination; booster evaluation was purely descriptive and conducted in a limited proportion of initially primed subjects. Co-administered with OPV at 2, 4 and 6 months of age, the new DTPw-HBV/Hib vaccine was found to be non-inferior to *Tritanrix*™-HBV/Hib in terms of seroprotection to the D, T, HBs and PRP vaccine antigens one month after completion of the 3-dose primary course. Immune response to all vaccine antigens was within the range previously reported for this or other similar DTPw-based vaccines [11-13,15-17,20].

Lot-to-lot consistency for the new DTPw-HBV/Hib vaccine was demonstrated, i.e., for anti-diphtheria, anti-tetanus, anti-HBs and anti-PRP, the standardized asymptotic 90% confidence intervals (CI) for the absolute difference between each pair of lots in the percentage of subjects seroprotected was within [-10%; 10%] and for anti-BPT, the 90% confidence interval for the ratio of geometric mean antibody concentrations (GMCs) between each pair of lots was within [0.5, 2] (data not shown). Based on these findings, data for the three vaccine lots used in this study were pooled for comparison against the control vaccine.

Antibody GMCs induced by the new DTPw-HBV/Hib vaccine following primary vaccination were observed similar to those induced by *Tritanrix™-*HBV/Hib, with the exception of anti-PRP GMC. However, almost all subjects achieved anti-PRP concentrations ≥1.0 μg/mL (≥97.3%) and post-primary anti-PRP GMCs were high (> 12.5 μg/mL) in both groups, suggesting that intergroup differences are unlikely to be of clinical relevance. A large body of evidence shows reduction of the PRP content of Hib conjugate-based vaccines to have no negative impact on either immune response or induction of immune memory to Hib [21-26]. Effective induction of immune memory was demonstrated with the reduced-content Hib component used in this DTPw-HBV/Hib vaccine [14,18]. In our study, a high degree of antibody persistence and a vigorous booster response was seen for all vaccine antigens including PRP in both groups, indicative of effective priming and induction of immune memory with both vaccines.

The new DTPw-HBV/Hib vaccine was found to have an acceptable reactogenicity profile. Some differences in reactogenicity were observed between the two vaccines. However, since very few subjects in either group sought medical advice for solicited symptoms it is likely that any differences had minimal clinical impact. Also, as no correction for multiplicity of endpoints was applied, statistically significant differences in reactogenicity should be interpreted with caution. Very few subjects in this study failed to receive all planned vaccine doses due to adverse events (2 in the DTPw-HBV/Hib group and 1 in the *Tritanrix*™-HBV/Hib group). Indeed, by eliminating the need for multiple injections, such combination vaccines are likely to promote compliance with infant immunization schedules [2-4]. In both groups, the incidence of solicited symptoms was higher after the booster dose than after the primary doses, as has previously been reported following booster vaccination with DTPw- and diphtheria-tetanus-acellular pertussis based vaccines [14,15,17,27,28].

## Conclusions

In conclusion, the development of new DTPw-based combination vaccines is essential for the success of current and future childhood mass vaccination programs in countries where resources are limited. Combining DTPw vaccines with HBV and Hib performs a key role in increasing vaccine coverage rates in line with WHO targets. Results of this study confirm the suitability of GSK Biologicals' new DTPw-HBV/Hib vaccine containing DTPw antigens from a new source and a reduced quantity of PRP as an alternative to currently licensed DTPw-based combination vaccines for use as a primary or booster dose in routine pediatric vaccination programs.

## Abbreviations

ATP: according to protocol; BPT: *Bordetella pertussis *toxin; CI: confidence interval; DTPw: Diphtheria-tetanus-whole-cell pertussis; ELISA: enzyme-linked immunosorbent assay; GMC: geometric mean antibody concentration; GSK: GlaxoSmithKline Biologicals; HBsAg: hepatitis B virus surface antigen; Hib: *Haemophilus influenzae *type B; HBV: hepatitis B virus; IU: international units; OPV: oral poliovirus vaccine; PRP: polyribosylribitol phosphate; SAE: serious adverse events; SD: standard deviation; WHO: World Health Organization

## Competing interests

This study was funded by GlaxoSmithKline Biologicals. JC, AC, ILF, J-MJ are employees of GlaxoSmithKline Biologicals or were employed at the time of the study.

All authors participated in the design or implementation, analysis and interpretation of the study, the writing of the report and the decision to submit the manuscript for publication.

## Authors' contributions

AC participated in the design of the study and performed the statistical analysis. FE, MT, AG, KA, JC, ILF, J-MJ conceived of the study, and participated in its design and coordination and helped to draft the manuscript. All authors read and approved the final manuscript.

## Pre-publication history

The pre-publication history for this paper can be accessed here:

http://www.biomedcentral.com/1471-2334/10/297/prepub
